# Induction of Innate Immune Memory by Engineered Nanoparticles in Monocytes/Macrophages: From Hypothesis to Reality

**DOI:** 10.3389/fimmu.2020.566309

**Published:** 2020-10-06

**Authors:** Paola Italiani, Giacomo Della Camera, Diana Boraschi

**Affiliations:** Institute of Biochemistry and Cell Biology (IBBC), National Research Council (CNR), Naples, Italy

**Keywords:** innate memory, monocytes, macrophages, nanoparticles, epigenetics, metabolism

## Abstract

The capacity of engineered nanoparticles to activate cells of the innate immune system, in particular monocytes and macrophages, is considered at the basis of their toxic/inflammatory effects. It is, however, evident that even nanoparticles that do not directly induce inflammatory activation, and are therefore considered as safe, can nevertheless induce epigenetic modifications and affect metabolic pathways in monocytes and macrophages. Since epigenetic and metabolic changes are the main mechanisms of innate memory, we had previously proposed that nanoparticles can induce/modulate innate memory, that is, have the ability of shaping the secondary response to inflammatory challenges. In light of new data, it is now possible to support the original hypothesis and show that different types of nanoparticles can both directly induce innate memory, priming macrophages for a more potent response to subsequent stimuli, and modulate bacteria-induced memory by attenuating the priming-induced enhancement. This evidence raises two important issues. First, in addition to overt toxic/inflammatory effects, we should consider evaluating the capacity to induce innate memory and the related epigenetic and metabolic changes in the immunosafety assessment of nanomaterials, since modulation of innate memory may be at the basis of long-term unwanted immunological effects. The other important consideration is that this capacity of nanomaterials could open a new avenue in immunomodulation and the possibility of using engineered nanomaterials for improving immune responses to vaccines and resistance to infections, and modulate anomalous immune/inflammatory reactions in chronic inflammatory diseases, autoimmunity, and a range of other immune-related pathologies.

## Introduction

In the last decades two important research fields have crossed paths: nanotechnology and immunology. On the one hand, nanotechnologies have experienced a huge development in many different application areas, including nanomedicine. Many nanoparticles (NP) have been engineered for appropriate modulation of their physical-chemical properties (e.g., nano-size, chemical composition, crystallinity, inorganic or organic functionalization, solubility, shape, aggregation behavior, electronic surface charge, and flexibility), usefulness for diagnostics (e.g., molecular imaging), and therapeutic (e.g., drug delivery system) purposes (“theranostic nanoparticles”) (1). On the other hand, the necessity to use NP that are safe for the organism has required to improve our knowledge on the interactions between NP and the immune system, in particular the impact on innate immunity (2, 3). Indeed, the innate immune system is the first line of defense of the organism, and the cells of the innate immune system are the first that interact with NP introduced into the body (3). As a result of this interaction, NP could either raise no response or trigger an innate/inflammatory reaction, based on whether or not the innate cells “sense” the particles as potentially dangerous. In nanomedicine, this is a crucial issue for two reasons: 1. to avoid an immune reaction against NP that could provoke damage in the body; 2. to avoid immune surveillance and clearance by the immune cells at the expense of the NP efficacy as diagnostic or therapeutic tools. Over the last years, the interaction between NP and the immune system has been widely investigated, and it is currently of great interest for the possibility of using immunomodulatory NP formulations in the therapy of a wide range of diseases. In fact, if on the one hand NP can directly activate undesirable immune effects (4), on the other hand they can be manipulated to attain reduction of their immunotoxicity (5) and tailored in order to get therapeutically desirable immunomodulation (i.e., immunostimulation or immunosuppression) (3, 6, 7).

It is well-known that invertebrates can adapt in virtually any ecosystem and survive infections by only relying on innate immunity (8). This implies the capacity to mount a faster and more effective response upon re-exposure to a stimulus (“immune training/priming”) although they do not have an adaptive immune system. This kind of improved secondary defensive response in invertebrates is considered an adaptive aspect of innate immunity and the demonstration of a *bona fide* immune memory. The same phenomenon has been observed in the innate immune system of mammals, in parallel to the much better-known immune memory displayed by the adaptive immune system. Indeed, both the innate and the adaptive immune system (concomitantly present in higher vertebrates) aim to improve their efficacy following a primary exposure to pathogens or other challenges (e.g., endogenous molecules, such as oxLDL, high level of glucose, uric acid, etc.) (9–11) by altering the type and magnitude of response to secondary threats toward stronger and more effective responses (“potentiation”) or weaker and less self-damaging reactions (“tolerance”) (12–14). The concept that innate immune cells can react differently to secondary challenges can be defined as innate immune memory. At variance with adaptive immune memory, the innate memory is non-specific, that is, the cells are more or less reactive regardless of the origin/nature of the secondary stimulus. The higher or lower reactivity is assessed in terms of production of inflammatory and anti-inflammatory factors, and a metabolic and epigenetic reprogramming of the cells seems to be the molecular mechanism underlying this functional plasticity/flexibility (15).

The establishment of innate immune memory has a profound impact on the effectiveness of human immune responses. Indeed, innate memory seems to provide an explanation for the heterologous effects of vaccines, as suggested in the case of human volunteers immunized with the BCG vaccine against *Mycobacterium tuberculosis* (attenuated *Mycobacterium bovis* strain Bacillus Calmette-Guérin bacteria), who became less susceptible to unrelated subsequent infections, possibly because of their potentiated and thus more robust innate immune response (16, 17). However, while innate memory is expected to generate improved defense to new challenges, it is possible that it could also cause detrimental effects in inducing and/or maintaining autoimmune and autoinflammatory diseases, as recently discussed elsewhere (18).

Thus, there will be a tremendous benefit in understanding 1) which metabolic and epigenetic changes could possibly be targeted to either promote or prevent innate memory and 2) which agents may initiate or modulate innate memory toward potentiation or tolerance, to be used as therapeutic tools. In this perspective, NP appear to be good candidates for the design of new therapeutic strategies for modulation of innate memory (19, 20). Indeed, NP can be tailored/functionalized to modulate innate memory responses and act as immunosuppressors in diseases with exacerbated immune response/inflammation (autoimmune disorders and auto-inflammatory diseases, atherosclerosis, multiple sclerosis, diabetes, etc.) or as immunostimulants in conditions with reduced/compromised immune responsiveness (e.g., certain types of cancer, sepsis, or infections). Thus, the ability of NP to modulate the molecular mechanisms involved in the development of innate memory would provide an avenue for improving the therapy of these diseases.

On this basis, we should revise our approach in examining the effects of NP on innate immune responses by including the effects on the establishment and modulation of innate memory. In nanosafety studies, this becomes particularly important for avoiding detrimental NP effect on human health, in particular in immune-related diseases. For instance, if NP induce a potentiated memory response in autoimmune patients or a tolerant response in immunosuppressed patients, this will exacerbate the patients' conditions. On the other hand, in the case of nanomedicine it would be particularly promising having the possibility to modulate precisely the immune memory so as to potentiate secondary responses in immunosuppressed patients and, *vice versa*, reduce reactivity in autoimmune conditions.

To date, nanoimmunosafety studies have paid little/no attention to the possible effects of NP on innate immune memory. While a large body of work has focused on the NP effects on innate immune cells (3), understanding the effects of NP beyond this primary response is still overlooked, although it would lead to a truly complete view of the consequences of NP exposure in humans (21). Three years ago, we hypothesized that NP could have effects on the induction or modulation of the innate memory of human mononuclear phagocytes (21). After 3 years, we can support our original hypothesis in light of new knowledge regarding the molecular mechanisms underlying the development of innate memory (i.e., the metabolic and epigenetic reprogramming of the cell) and regarding the NP effects on the metabolism and the epigenetic modifications in innate immune cells (in particular, monocytes/macrophages). If NP can have an effect on the metabolism and can induce epigenetics changes, and if the molecular mechanisms involved in the development of innate memory are indeed metabolic and epigenetic changes, for transitive property we suppose that NP could have an effect on innate memory, and therefore this effect should be taken into account in the evaluation of the immunosafety/toxicity of NP. Moreover, we assume that the development of innate memory is not only restricted to microbial agents or endogenous molecules as mentioned before but also to non-biological stressors such as NP. The objective/focus of our review is to summarize the data supporting our deductive reasoning and to support the hypothesis with the very first experimental evidence of a direct effect of NP on the induction of innate memory in human monocytes/macrophages.

## Innate Memory and Epigenetic Reprogramming

Epigenetics is referred to as “a stably heritable phenotype resulting from changes in a chromosome without alterations in the DNA sequence” (22). Epigenetic modifications involved in development of innate memory are essentially three: DNA methylation, post-translational modifications of histones, and regulation of non-coding RNAs (ncRNAs), that is, short (miRNA) and long (lncRNA) RNA. All of them are involved in the regulation of gene expression.

Innate immune memory shows as an enhanced or reduced reactivity of innate immune cells upon a second challenge, which mostly includes an increased or decreased production of inflammation-related factors. This production depends on the coordinated regulation of expression of an array of genes, pointing at the importance of epigenetic mechanisms in the establishment of innate memory. The epigenetic reprogramming of innate cells in innate immune memory has been extensively reviewed elsewhere (15, 23). Here, we briefly report the main epigenetic marks identified so far in blood monocytes and bone marrow-derived macrophages, the innate cells that are the best-known effectors in the context of innate immune memory.

A high level of trimethylation of histone 3 lysine 4 (H3K4me3) on gene promoters is generally associated with a robust transcription uniformly across various cell types (24, 25). In the case of human monocytes primed *in vitro* with β-glucan from *Candida albicans*, it has been observed that cells present an accumulation of H3K4me3, acetylation of histone 3 lysine 27 (H3K27ac), and monomethylation of histone 3 lysine 4 (H3K4me1) on enhancers of immune genes. Upon removal of the priming stimulus, H3K27ac was lost over time, but H3K4me3 remained on the chromatin ensuring a potentiated immunological response to a second stimulation. H3K4m1 was also maintained over time, although not all genome regions retained this mark (26–28). The same marks have been observed in murine hematopoietic progenitors (29) stimulated with β-glucan *in vitro*, in myeloid progenitors and bone marrow-derived macrophages from BCG vaccinated mice (30), and in monocytes isolated from healthy volunteers after vaccination with BCG (31), supporting a role for these specific modifications in the epigenetically regulated establishment of innate memory. These epigenetic changes allow the transcriptional machinery to access DNA thereby promoting an enhanced transcription of inflammatory genes such as TNF-α and IL-6 (31). Thus, these epigenetic marks appear to be associated to a particular type of memory, that is, the potentiation of the secondary response. Conversely, it has been proposed that the lack (or delayed establishment) of the same epigenetic changes after cell priming with lipopolysaccharides (LPS) from *Escherichia coli* could be responsible of the other type of innate memory, that is, tolerance (27). This is further suggested by the finding that β-glucan could increase H3K27ac on enhancers of LPS-tolerized genes along with the re-establishment of the macrophage capacity to produce inflammatory cytokines after a re-exposure to LPS. These findings also highlight the possible reversibility of innate memory phenotypes (27). Moreover, H3K4me3 is selectively present only on promoters of genes that maintained an active expression (non-tolerized genes) during LPS tolerance induced in murine macrophage (32).

To date, the role of DNA methylation in innate immune memory is poorly understood. A distinct methylation pattern was observed in *in vitro* LPS-primed human monocytes after 6 days from stimulation, while β-glucan-treated cells present a methylome similar to that of naïve macrophages (27).

Likewise, the contribution of miRNAs to the development of innate memory remains largely unexplored. A recent study reported how specific miRNAs can regulate tolerance in murine macrophages *in vitro*. The authors identified miR-221 and miR-222 as regulators of the functional reprogramming of mouse bone marrow-derived macrophages during LPS-induced tolerance *in vitro*. Prolonged stimulation with LPS leads to increased expression of miR miR-222, which downregulates the *Brg1* gene and provokes the silencing of a subset of inflammatory genes through SWItch/Sucrose Non-Fermentable (SWI/SNF) and STAT-mediated chromatin remodeling. The involvement of miR-221 and miR-222 is confirmed also in patients with sepsis, with an increase in their expression in peripheral blood mononuclear cells correlated with immunosuppression and organ damage (33).

lncRNAs have only recently started to be investigated in the context of innate memory (34, 35). lncRNA seems to facilitate the H3K4me3 epigenetic priming in innate immune cells, such as monocytes (34). 3D chromatin topology can place lncRNAs proximal to innate immune gene promoters, and, through formation of lncRNA/protein complexes, lncRNAs can regulate gene transcription by influencing the access of transcription-regulating proteins to their target genes. Indeed, recent data indicate that genes encoding inflammatory cytokines (e.g., IL-1β, IL-6) and chemokines (e.g., IL-8, CXCL1, 2, 3) engage in chromosomal contacts with a special type of lncRNAs, the immune-gene priming lncRNAs (IPLs). For example, one of these IPLs, UMLILO, acts in cis (i.e., on the same molecule of DNA as the genes to be transcribed) and points the adapter protein WDR5 and the histone methyltransferase MLL1 complex to the inflammatory gene promoters thereby enabling their H3K4me3 epigenetic priming, prior to their transcriptional activation, an event that leads to an enhanced transcription of the immune genes in response to a secondary challenge (36).

Recently, it has been also speculated that another type of lncRNA, the enhanced RNAs (eRNAs), may play a role in memory immune responses (15). eRNAs derive from the transcription of enhancers, are cell-specific, and are involved in the regulation of chromosomal looping and in the transcription of tissue-specific genes/contacts between target genes and enhancers. For example, eRNAs can interact with mediator complex or Yin Yang 1 to regulate chromosomal contacts between target genes and enhancers (37, 38). Also, eRNAs can interact with proteins such as p300 and CBP (cyclic adenosine monophosphate response element-binding protein), able to stimulate catalytic histone acetyltransferase activity at specific genomic loci (39). As a consequence, the gene transcription is activated. Therefore, eRNAs may play a role in innate memory responses by regulating looping at key enhancers and histone acetylation. Moreover, latent enhancers (a sub-class of enhancers), upon LPS stimulation, acquire the typical histone modifications (H3K4me1 and H3K27Ac) associated with an active enhancer region and that persist after the removal of the stimulus in mouse bone marrow-derived macrophages (40).

## Innate Memory and Metabolic Reprogramming

Along with epigenetic changes, another hallmark characteristic associated with the induction of innate memory is the metabolic reprogramming of innate cells.

Changes in cellular metabolism are associated with different immune functions (41) and macrophage functional phenotypes (42). In the case of mononuclear phagocytes, activated monocytes and classically activated macrophages (M1) are characterized by an enhanced glycolytic metabolic state for ATP production, an activated pentose phosphate pathway (PPP), an impaired oxidative phosphorylation (OXPHOS), and an anabolic repurposing of metabolites of the tricarboxylic acid cycle (TCA), with pyruvate fermented in lactate. On the other hand, naïve quiescent monocytes and alternatively activated (IL-4-induced) macrophages (M2) preferentially make use of OXPHOS for ATP production, while the concomitant presence of glycolytic pathways generate the pyruvate for fueling the TCA cycle.

Human monocytes stimulated *in vitro* with β-glucan or BCG show a metabolic shift similar to that observed in classically activated macrophages (43, 44), that is, elevated consumption of glucose by glycolysis, conversion of pyruvate to lactate, and decreased OXPHOS with reduced oxygen consumption (Warburg effect). In both cases (BCG- and β-glucan-primed monocytes), the activation of the Akt-mTOR-HIF1α pathway drives the shift from OXPHOS to aerobic glycolysis (43, 44). Moreover, in β-glucan- and BCG-primed monocytes the metabolites of TCA cycle (citrate, succinate, malate, fumarate, and 2-hydroxyglutarate) are increased probably thanks to increased glutamine metabolism (glutaminolysis) (43, 45). Glutamine is metabolized first into glutamate and then in α-ketoglutarate and can become a source of succinate, fumarate, and citrate, which in turn replenish the TCA cycle (46). Furthermore, β-glucan-primed monocytes show an up-regulation of genes involved in the cholesterol biosynthesis. The activation of the cholesterol biosynthesis pathway, but not its actual synthesis, is also involved in the metabolic reprogramming of primed cells, as it has been shown that blocking mevalonate (a metabolite of the cholesterol synthesis pathway) with statins prevents the induction of innate memory (47). Although the fatty acid synthesis also increases in macrophages upon LPS stimulation, the blockade of this synthesis does not affect the memory phenotype in monocytes primed with β-glucan and restimulated with LPS (44).

Recently, it has also been shown that the liver X receptor (LXR), a key regulator of cholesterol and fatty acid homeostasis, may play a role in the establishment of innate memory (48). Human monocytes primed *in vitro* with LXR agonists and challenged after 5 days with a TLR2 agonist, Pam3Cys, showed an inflammatory activation accompanied by epigenetic reprogramming (increased H3K27ac and H3K4me3 on IL-6 and TNF-α promoters) and metabolic reprogramming (increased lactate production and decreased oxygen consumption) with respect to unprimed cells. Moreover, the authors observed an increase, after priming with LXR agonists, in the expression of several genes involved in the synthesis and metabolism of acetyl-CoA and that acetyl-CoA is able *per se* to prime monocytes and induce a memory phenotype upon challenge with TLR2 agonist. All these effects depend on both mevalonate pathways and IL-1β signaling. In fact, the inflammatory memory phenotype induced by priming with LXR agonists is attenuated by inhibition of mevalonate pathways with fluvastatin, an agent that competitively inhibits hydroxymethylglutaryl-coenzyme A (HMG-CoA) reductase, which in turn catalyzes the conversion of HMG-CoA to mevalonic acid, the rate-limiting step in cholesterol biosynthesis. Likewise, blocking of IL-1β with its receptor antagonist, IL-1Ra, has the same inhibitory effect.

Metabolic changes in blood monocytes or macrophages during sepsis, in mice and humans, mirror the same shift toward aerobic glycolysis during the hyperinflammatory phase and a decreased OXPHOX and TCA cycle during the hypoinflammatory phase (49). These aspects are extensively reviewed elsewhere (50).

Furthermore, sepsis is able to induce an inflammatory memory phenotype in bone marrow monocytes. Indeed, post-septic naïve bone marrow monocytes and blood monocytes isolated from mice showed an increased cytokine production when stimulated with LPS *in vitro* (51).

## Interplay Between Epigenetic and Metabolic Reprogramming

As mentioned above, the epigenetic reprogramming involved in the development of innate memory encompasses mainly methylation and acetylation of histones and the activity of lncRNA, while the metabolic reprogramming results in changes to glycolysis, TCA cycle, glutaminolysis, and cholesterol metabolism. Different genes involved in these pathways are quickly upregulated upon stimulation, causing changes in the levels of intracellular metabolites. The metabolic reprogramming and epigenetic reprogramming are strictly linked because the former supplies the metabolites and co-factors critical for the induction and preservation of the latter in the establishment of a memory phenotype. This immunometabolic cross-talk seems to be a common denominator across species, from nematodes to vertebrates, and the two reprogramming may represent ancestral and conserved mechanisms underlying the development of innate memory (52). The cross-talk between metabolism and epigenetics occurs because the stimulation of innate immune cells with microbial or endogenous ligands modifies the epigenetic landscape of metabolic regulators. Indeed, the genes involved in the metabolism of glucose, glutamine, and cholesterol and in the TCA cycle pathway are upregulated after stimulation, causing changes in the levels of intracellular intermediates (36). These various metabolites/intermediates in turn act as co-factors or can influence the functionality of the enzymes responsible for epigenetic modifications (53, 54), as illustrated in the following examples. Metabolic intermediates, such as acetyl-CoA produced from glycolysis and glutaminolysis, act as donors of acetyl groups for histone acetylation (55). An intermediate of the TCA cycle, α-ketoglutarate, is important for the functions of ten-eleven translocation (TET) proteins involved in DNA methylation (56) and consequently involved in β-glucan- and BCG-induced long-term reprogramming of myeloid progenitors in the mouse bone marrow (29, 30) and is also a cofactor of the JmjC domain-containing histone demethylases (KDMs) (57). S-adenosyl methionine is a co-substrate of histone and DNA methyl transferases, being the donor of methyl groups (58). Succinate and fumarate can inhibit demethylation reactions (56), and in particular fumarate (derived from glutaminolysis for the replenishment of intermediates of the TCA cycle) inhibits the H3K4 demethylase KDM5, favoring the enrichment of H3K4me3 at the promoters of inflammatory genes (44). Furthermore, succinate, fumarate, and mevalonate stabilize HIF1α, thereby sustaining Akt-mTOR-HIF1α signaling (43, 46) that, as mentioned before, is responsible of the switch from oxidative phosphorylation to aerobic glycolysis in human macrophages.

These few examples underline the complex and intimate relationship between the cellular metabolic machinery and the epigenetic modulators. Exhaustive details on such relationship are reported elsewhere (15, 42, 53).

## Nanoparticles and Epigenetics

The effects of engineered NP and nanomaterials on epigenetic modifications has been extensively and excellently reviewed (59–63). It is quite clear that NP can be considered potential epimutagens (although a lot still remains to be unknown). Thus, in the evaluation of NP safety, epigenetic effects should be listed among potential health hazards, in addition to genotoxic and cytotoxic effects (64, 65). Most studies on the epigenetic effects of NP have been carried out *in vitro* on human tumors or transformed cell lines (e.g., A549, HaCaT, MCF-7, BEAS-2B, HepG2, 293T, and GLC-82) and *in vivo* in mice or on exposed subjects/workers. Epigenetic changes induced by NP can vary depending on NP characteristics such as size, shape, chemical structure, surface functionalization, exposure dose, short-term or long-term exposure, and type of cells.

We refer to aforementioned literature for a broader vision and more detailed reading on the NP effects on epigenetic changes, while here we briefly report work that directly or indirectly pertains to the innate immune system. Although many studies describe cytotoxicity induced by NP for several cell types, no data are available that could link NP-induced toxicity to epigenetic alterations.

There is evidence that some NP can enter the nucleus and interact with positively charged core histones, as it has been proven for anionic Cd-Te quantum dots (QD). The QD enter the nucleus of THP-1 cells, where they preferentially bind to core histones as opposed to other nuclear macromolecules, such as DNA and RNA, and change their physical-chemical properties leading to an increased formation of QD/protein aggregates (66).

Among epigenetic modifications, NP can induce both global DNA methylation and methylation at the level of specific genes. Notably, NP-induced DNA methylation can differ depending on NP and cell type of NP and cells exposed. For example, a study has investigated the effects of the exposure to NP of copper oxide (CuO) or titanium dioxide nanoparticles (TiO_2_) on the cellular epigenome of human and murine macrophage-like tumor cells (THP-1 and RAW264.7, respectively) (67). In particular, the authors have addressed the methylation status of the two most abundant transposable elements (TE) in the human genome (LINE1 and Alu) and in the mouse genome (SINEB1 and SINEB2). They observed that CuO NP induced hypermethylation in LINE1 and Alu elements in THP-1, while CuO and TiO_2_ NP enhanced the methylation level of SINEB1 in RAW264.7.

In another study, despite no significant difference in global DNA methylation, it was possible to observe DNA methylation changes in the promoter CpG regions of genes of enzymes involved in the epigenetic regulation, DNA repair (i.e., DNMT1, HDAC4, ATM), and gene of TGF-β repressor (SKI) in blood cells of workers exposed to multi-walled carbon nanotubes (MWCNT) with respect to unexposed control individuals (68). Furthermore, workers exposed to particulate matter and polycyclic aromatic hydrocarbons exhibited, in their peripheral blood leukocytes, significantly higher average levels of methylation at the promoter of tumor-suppressor genes, such as CDKN2A, APC, and MLH1, and significant hypomethylation at the repetitive DNA sequence LINE-1 (a sequence involved in DNA folding/packaging) in comparison to unexposed workers (69).

Another study showed DNA methylation changes in human monocyte-like cells (THP-1) after incubation with either single-walled or multi-walled CNT. By assessing methylation of single CpG sites, it was observed that CNT induced gene-specific differential methylation, with promoter hypomethylation evident for a thousand different genes, compared with the control samples. Some of these genes were associated with alterations in some signaling pathways, such as the PI3K-AKT-mTOR, JAK-STAT, MAP, and VEGF pathways, and platelet activation. The authors discussed the possible contribution of these epigenetic alterations on genes involved in macrophage polarization and hypothesized a mixed M1/M2 macrophage functional phenotype upon CNT exposure (70).

Using the LUminometric Methylation Assay (LUMA) and the 5-methylcytosine (5-mC) quantification assay, Brown et al. (71) elucidated the effect of oro-pharyngeal instillation of MWCNT on global DNA methylation and specifically on genes associated with inflammation (TNF-α and IFN-γ) and with fibrosis (Thy-1) in C57BL/6 mice. They found that MWCNT leads to DNA hypomethylation at the inflammatory gene promoters and hypermethylation at the fibrotic gene promoter in the lung and to a reduction of global DNA methylation in lung and circulating white blood cells, 7 days post-exposure, which coincided with development of MWCNT-induced fibrosis.

More research is needed to establish a specific role for dysregulated ncRNA upon *in vivo* and *in vitro* exposure to NP. Among the few data available, a recent study has shown that miR-350 may promote apoptosis in RAW264.7 cells through the negative regulation of the PIK3R3 gene and that exposure to TiO_2_ NP caused an increase in miR-350 and a decrease in PIK3R3 (72).

Another study investigated significant changes in mRNA and ncRNA expression profiles in the blood of workers exposed to MWCNT compared to non-exposed group. Gene set enrichment analysis and pathway analysis showed that differentially expressed sets of miRNAs and their target genes were mainly involved in cell cycle regulation/progression/control, apoptosis, and proliferation and revealed the potential of MWCNT to trigger pulmonary effects and carcinogenic outcomes in humans (73).

Interestingly, upon exposure to silver, titanium dioxide, and zinc oxide NP, differentiated macrophage-like THP-1 cells presented a profile of miRNA and miRNA variants, called isomiRs, that was unique to each NP type, and an identified co-regulated miR-mRNA cluster (encompassing hsa-miR-142-5p,−342-3p,−5100,−6087,−6894-3p, and−7704) seems to be a potential biomarker of metal-based nanoparticle exposure (74).

## Nanoparticles and Cell Metabolism

While the effects of NP on monocytes/macrophages activation have been widely investigated, those on cellular metabolism is still poorly explored (75). Here we will focus on some evidence of the NP effects on metabolic pathways of human or murine monocytes and macrophages extrapolated from activation/cytotoxicity studies. Chen et al. (76) reported metabolic dysfunctions in macrophages exposed to TiO_2_ NP. In particular, the authors observed that both the levels of ATP and the metabolic flux at the level of most TCA metabolites were attenuated in a dose-dependent manner in RAW264.7 and bone marrow-derived macrophages (BMDM) after 24 h exposure to NP. These results suggest that TiO_2_ NP could cause a significant mitochondrial dysfunction in macrophages, through the down-regulation of the TCA cycle and ATP production.

In another study, cells of mouse macrophage-like line J774A.1 were exposed to a high dose of silver NP for 24 h, and the NP effects have been evaluated immediately after treatment (24 h) and after a recovery period of 72 h after NP removal (77). The authors observed that, although specialized macrophage functions (e.g., phagocytosis) are restored during the recovery period, lipopolysaccharide-induced cytokine (e.g., IL-6 and TNF-α) and nitric oxide production, some enzymatic activities involved in the TCA cycle (e.g., NADPH-dependent isocitrate dehydrogenase and malate dehydrogenase) and glucose consumption, did not return to basal level, showing that some effects of silver NP persist after cessation of exposure.

Another study on the murine macrophage RAW264.7 cell line showed that exposure to silk, poly(lactic-co-glycolic acid) (PLGA), and silica NP (considered good candidates for nanomedicine applications) induced some metabolic changes that were independent of the NP type; that is, higher glucose consumption and lactate production (indicative of increased glycolytic activity), high level of itaconate and succinate (two intermediate metabolites of TCA cycle), decreased amino acids levels (e.g., aspartate and glutamate), and ATP decrease over time. Other metabolic changes were specific for individual NP type, such as glutaminolysis (reduced or enhanced or unchanged based on NP types) and a variation of the creatine kinase/phosphocreatine pathway (enhanced or unchanged based on NP types) (78).

In another study on murine macrophage-like RAW264.7 cells, the cell metabolic profiles have been evaluated upon exposure to unmodified or PEGylated silk fibroin NP (another promising nanomedicine) (79). Exposure to unmodified NP caused an increase in glycolysis and reprogramming of the TCA cycle (with a significant increase in lactate, itaconate, and succinate), an increase in the creatine kinase/phosphocreatine pathway, a decrease in ATP level and in the levels of several amino acids (aspartate, glutamine, glutamate, and alanine) and an increase in others (glycine, lysine, branched chain, and aromatic amino acids), and an increase in cholesterol and phosphatidylcholine together with decrease in unsaturated fatty acids. By contrast, milder effects were observed for all these metabolic pathways in macrophages stimulated with PEGylated silk fibroin NP. Thus, NP appear to have a direct effect on the metabolism of innate immune cells. In addition, there is evidence that NP can also modulate cell metabolism by affecting/interfering with biological processes such as autophagy and differentiation/polarization.

For example, Wu et al. (80) demonstrated that dextran-coated superparamagnetic iron oxide NP (Dex-SPION) induced autophagosome accumulation in human monocytes. By inhibiting the autophagy, they observed increased production of inflammatory cytokines (TNFα, IL-1β, and IL-6) upon exposure to Dex-SPION, suggesting that the induction of autophagy by these NP has an important role in the modulation of human monocytes' function. Autophagy as a highly conserved metabolic process aiming to sequester (in autophagosomes) and digest (by lysosomal pathways) injured organelles and proteins in all eukaryotic cells (81) and is strictly linked to metabolic changes during monocyte differentiation and macrophage polarization (82). Although underlying mechanisms are still debated, it seems that autophagy is strictly linked to the metabolic changes during immune cell differentiation, and autophagy and metabolism are both regulated through AMPK and mTOR signaling cascades (82). This would imply that NP effects on autophagy will impact cell metabolism.

Many studies reveal an immunomodulatory role for NP (e.g., Au NPs, SiO_2_ NP, functionalized polystyrene, and molybdenum disulfide quantum dots) that can drive macrophages polarization toward M1- or M2-like phenotypes (83–85). M1-like cells are classically activated inflammatory macrophages mainly involved in host defense to infections and tumors, while M2 defines alternatively activated macrophages mainly involved in type 2 inflammation, anti-inflammatory responses, wound-healing, tissue remodeling, and tumor growth (86–89). Considering the significant metabolic differences between M1 and M2 macrophages, the effects of NP on macrophage polarization should be considered in terms of their possible effects in inducing metabolic reprogramming and, accordingly, in the development of a memory phenotype in these cells.

A proven relationship between metabolism and cellular redox state also exists (90, 91). Innate immune cells, especially macrophages and neutrophils, produce toxic reactive oxygen and nitrogen species (ROS and RNS). As an example, when circulating monocytes migrate into inflamed or infected tissues, they differentiate in M1 macrophages that release superoxide anion radicals, nitric oxide, and hydrogen peroxide (H_2_O_2_) to support pathogen killing (oxidative burst). The production of ROS requires a rapid burst of energy (glycolysis and rapid availability of NADPH), and, in turn, ROS production is required for the switch from oxidative phosphorylation to glycolysis (for details refer to 90). However, an excessive accumulation of ROS and NOS (oxidative stress) can be dangerous also for macrophages and other surrounding host cells, and, if maintained over time, it can lead to oxidation of proteins, lipids, carbohydrates, and DNA, causing metabolic dysfunction and DNA damage. The oxidative modifications of these molecules may provoke a re-programming of monocytes/macrophages. For example, an oxidative modification, S-glutathionylation (reaction between oxidized thiol group of protein and glutathione for the formation of mixed disulfides), occurs in proteins of both human monocytic cell line (THP-1) stimulated with H_2_O_2_ and after metabolic stress due to low-density lipoprotein (LDL) and high glucose exposure and in peritoneal macrophages of mice exposed to long-term metabolic stress. This modification alters the expression and function of proteins involved in the metabolism and inflammatory functions of monocytes/macrophages (92). ROS can provoke DNA damage directly or influence the methylome causing oxidative DNA lesions and can indirectly affect epigenetic mechanisms. These alterations are frequently associated to aging and immunosenescence (93–95).

These scenarios offer other levels at which NP can affect innate memory. NP can induce oxidative stress, and, as a consequence, ROS can oxidize enzymes involved in metabolism and lead to DNA damage. There is abundant evidence in the literature on the capacity of different NP to modulate ROS generation in innate immune cells. For example, SiO_2_, Au, Fe_x_O_y_, and carbon-based NP increase ROS production in macrophages (96–100). Moreover, it is worth noting that DNA damage due to NP-mediated oxidative stress in blood cells could be particularly important in age-related hematologic abnormalities. For example, it could affect TET2, one of the genes involved in clonal hematopoiesis, whose mutations are associated with increased risk of hematologic cancer and cardiovascular diseases (101, 102). The effects of NP on oxidative stress have been well-investigated and reviewed elsewhere (103–105).

The role of NP-induced ROS in the functional re-programming of immune cells toward a memory phenotype has not been investigated yet, despite its expected relevance.

These few studies, limited in number and mostly targeting transformed murine cells, nevertheless provide evidence that NP can have an effect in the metabolic reprogramming of monocytes/macrophages. The metabolic effects of NP should be thoroughly addressed, in future studies, especially because even NP that are considered non-toxic might have long-term effects on cellular metabolism and energy homeostasis (75).

Table 1 summarizes the main effects of NP on epigenetic and metabolic reprogramming of innate immune cells, macrophage polarization, and ROS/RNS production.

**Table 1:** Main effects of NP on epigenetic and metabolic reprogramming of innate immune cells.

**Nanoparticles**	**Cells**	**Overall effect**	**References**
**Epigenetic reprogramming**			
QD	THP-1	Binding to core histones and formation of QD/protein aggregates	(66)
CuO, TiO_2_	THP-1, Raw264.7	Methylation of transposable elements	(67)
MWCNT, PAH	Human blood cells	Methylation at the promoter of genes involved in tumor suppression, DNA repair	(68, 69)
SWCNT	THP-1	Hypomethylation in genes associated to signaling pathways and macrophage polarization	(70)
MWCNT	Murine blood cells	Hypomethylation in genes associated with inflammation	(71)
TiO_2_	Raw264.7	miR-350 increase	(72)
MWCNT	Human blood cells	Changes in mRNA and ncRNA expression profile	(73)
TiO_2_, ZnO	THP-1	Changes in miRNA profile	(74)
**Metabolic reprogramming**			
TiO_2_	Raw264.7, BMDM	Reduction of ATP and TCA metabolites, mitochondrial dysfunction	(76)
AgNP	J774A.1	Modulation of TCA enzymes and glucose consumption	(77)
Silk, PGLA, silica	Raw264.7	Increase in glycolysis and TCA intermediates, decrease in amino acids and ATP. Modulation of glutaminolysis and creatine kinase/phosphocreatine system	(78)
Silk fibroin	Raw264.7	Increase in glycolysis, TCA intermediates, creatine kinase/phosphocreatine system, modulation of amino acid levels, increase in cholesterol, decrease in unsaturated fatty acids	(79)
Dex-SPION	Human monocytes	Induction of autophagy	(80)
**Macrophage polarization**			
PS-COOH, PS-NH2	MDMs	skewing of M2 polarization	(83)
TPP-MoS2 QDs	Microglia	Switching from M1 to M2	(84)
Peptide-coated AuNP	BMDMs	M2 polarization	(85)
AgNP, AuNP	TAMs	Switching from M2 to M1	(97)
SPIONs	BMDMs, THP-1	M1 polarization	(98)
Iron oxide	RAW264.7, BMDMs	M1 polarization	(99)
MWCNT	RAW264.7	M1/M2 mixed status	(100)
**Oxidative stress**			
Silica	Peritoneal macrophage, RAW264.7	ROS/RNS increase	(96)
AuNP	TAMs	ROS/RNS increase	(97)
SPIONs	BMDMs, THP-1	ROS/RNS increase	(98)
Iron oxide	RAW264.7, BMDMs	ROS increase	(99)
MWCNT	RAW264.7	ROS increase	(100)

*QD, Cd-Te quantum dots; MWCNT, multi-walled carbon nanotubes; SWCNT, single-walled carbon nanotubes; PAH, polycyclic aromatic hydrocarbons; PGLA, poly lactic-co-glycolic acid; SPIONs, superparamagnetic iron oxide nanoparticles; Dex-SPION, dextran-coated superparamagnetic iron oxide nanoparticles; PS-COOH and PS-NH2, carboxyl-(PS-COOH) and amino-functionalized (PS-NH2) polystyrene nanoparticles; TPP-MoS2 QDs (3-carboxypropyl)triphenyl-phosphonium bromide-conjugated 1,2-distearoyl-sn-glycero-3-phosphoethanolamine-N-[amino(polyethylene glycol)-2000]-functionalized molybdenum disulfide quantum dots; MDMs, human monocyte-derived macrophages; BMDMs, bone marrow-derived macrophages; TAMs, tumor-associated macrophages*.

Most studies investigating the immune effects of NP on epigenetics and metabolism are performed using *in vitro* models based on human or murine cancer or immortalized cell lines. Cell lines have the advantages of easy handling and high reproducibility, but they have the disadvantage to be poorly predictive of human primary cell response (due to physiological and metabolic differences with normal primary immune cells) (106, 107), and, obviously, they often do not fully represent what occurs *in vivo*. In the use of primary murine cells there are important differences between primary murine and human immune cells, for example, differences in LPS-induced immunometabolism (108) or in inducibility of expression of genes involved in the inflammatory response (109). Thus, the use of blood isolated from subjects exposed to NP, and the use of primary immune cells isolated from healthy donors and stimulated *in vitro* with NP, remains the preferred choice, despite the limits due to the lack of *in vivo* system complexity and donor variability. However, in case of innate memory, the donor variability due to the individual history of previous infections/exposure is a critical issue for understanding the need for a personalized nano-immunotoxicological assessment.

## Nanoparticles Can Induce and Modulate Innate Immune Memory

**Figure 1:**
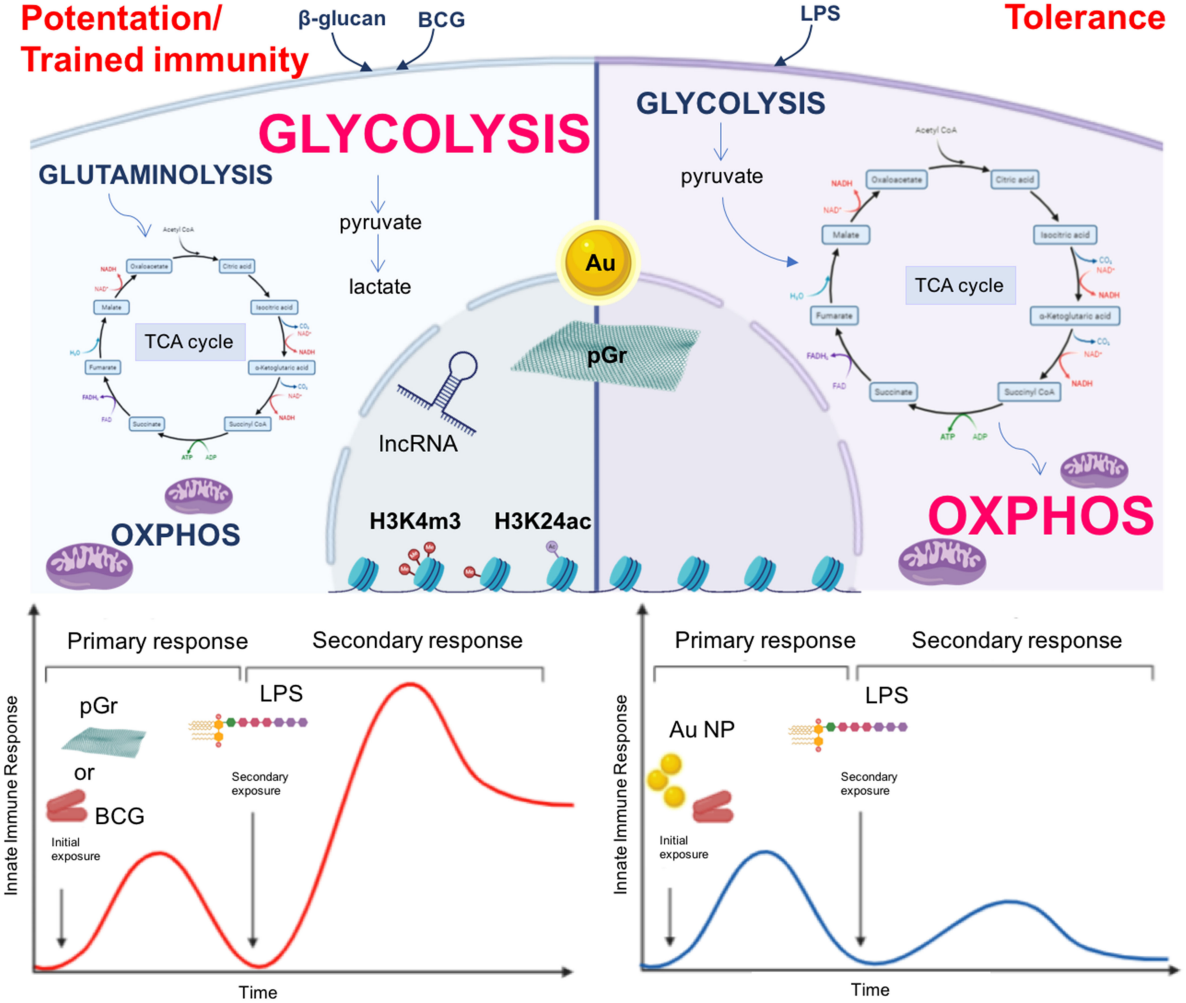
Nanoparticles as inducers of innate immune memory. Upper part: β-glucan or BCG-primed monocytes (left) have an enhanced glycolytic metabolic state and an impaired oxidative phosphorylation (OXPHOS). The tricarboxylic acid cycle (TCA) is fueled by metabolites derived from glutaminolysis, while pyruvate is mainly fermented into lactate. On the other hand, LPS-primed cells (right) preferentially use OXPHOS, and the glycolytic pathway generates pyruvate for fueling the TCA cycle. The epigenetic reprogramming occurring in β-glucan- and BCG-primed monocytes mainly encompasses histone methylation and acetylation and involves lncRNA, modifications that are lacking (or established late) in LPS-primed monocytes. NP (Au, pGr) may play a role in the induction and modulation of innate immune memory by regulating epigenetic and metabolic reprogramming of innate immune cells (dashed arrows). Lower part: BCG-primed monocytes (left) show an increased reactivity after a subsequent exposure to LPS. When monocytes are primed with BCG in the presence of Au NP, they show a reduced reactivity (right). Thus, Au NP are able to reduce the BCG-induced memory response in human primary monocytes by shifting the memory effect from potentiation/trained immunity to tolerance. By contrast, pGr (left) is able to prime directly murine BMDM, which responds to a subsequent LPS challenge with a potentiated reaction.

In light of what is discussed and reviewed so far, it is reasonable to speculate that NP may be able to induce and modulate innate immune memory by regulating epigenetic and metabolic reprogramming of innate immune cells, thereby influencing their ability to react to a second exposure (Figure 1, upper part). Indeed, recent evidence shows that this is the case. This evidence comes from NP of interest for biomedical applications, that is, gold (Au) NP and pristine graphene (pGr). As reported in our previous publication (21), preliminary studies show that Au NP could modulate the memory induced by LPS priming in human primary monocytes *in vitro* and that this response could differ in different individuals, likely due to individual immunological history of previous infections. More recently, we have shown that Au NP have a significant effect in reducing the BCG-induced memory in human primary monocytes *in vitro*. At variance with the tolerance induced by LPS priming, monocytes primed with BCG showed an enhanced production of inflammatory (IL-6 and TNF-α) and anti-inflammatory cytokines (IL-1Ra and IL-10) when challenged with LPS. Although Au NP did not have a direct effect in inducing a memory response *per se*, when monocytes were primed with BCG and Au NP together, a reduction in cytokine production in response to LPS challenge could be observed, compared to that in cells primed with BCG alone, suggesting that Au NP interfered with the induction of memory by BCG and shifted the memory response of monocytes toward a tolerant phenotype (110) (Figure 1, bottom part). This study provides the first evidence that Au NP can influence the development of innate immune memory triggered by microbial molecules or vaccines and affect the monocyte response to subsequent challenges.

In another recent study, Lebre et al. (111) proved that pGr can directly prime mouse BMDM *in vitro*. BMDM exposed to pGr for 24 h and, after 5 days, stimulated with LPS produced significantly higher amounts of the inflammatory cytokines IL-6 and TNF-α and lower amounts of the regulatory cytokine IL-10 compared to unprimed cells (Figure 1, bottom part). Challenge with other Toll-like receptor (TLR) agonists, such as CpG (TLR9 agonist) or the R848 (TLR7/8 agonist), yielded the same memory response in pGR-primed macrophages as that obtained with LPS, that is, enhancement of IL-6 secretion and decrease in IL-10 secretion. The induction of memory by pGr occurred through epigenetic reprograming, since preincubation of BMDM with inhibitors of histone methyltransferase or histone demethylase abolished the priming effect of pGr in terms of enhancement of LPS-induced IL-6 production. Interestingly, the same inhibitors did not have effect on LPS-induced IL-10 and TNF-α, suggesting a different regulatory mechanism for each inflammatory and anti-inflammatory factor.

Collectively, these data provide evidence that NP are able to reprogram macrophages to respond differentially to a second stimulation. However, while pGr can apparently induce innate memory *per se*, Au NP do not have a direct effect but can modulate the memory induced by another stimulus.

The molecular mechanisms by which Au NP affect innate memory induction have not been fully investigated yet, but it is reasonable to hypothesize that they can involve metabolic reprogramming more than epigenetic changes.

## Conclusions

In the last decades, engineered NP have found wide application in medicine, as diagnostic and therapeutic tools. As a consequence, the evaluation of nanoimmunosafety has become crucial in order to avoid unwanted immune system activation, which could contribute to chronic inflammation, autoimmunity, or allergy. Conversely, the ability of NP to interact with innate immunity could be exploited and, through rational engineering, directed toward an intentional enhancement or suppression of immune reactions to treat a range of disease conditions and in preventive vaccination strategies. The recent evidence that innate memory exists also in vertebrates and that it is based on the epigenetic and metabolic reprogramming of innate cells is opening a new area of investigation in the assessment of NP immune interactions. Some studies have shown that NP can induce epigenetic modifications (DNA methylation, histone modification, and non-coding RNA modulation) and metabolic changes in monocytes and macrophages, the major innate immune cells. Other studies have revealed that certain types of NP can induce innate memory or modulate the memory induced by bacterial priming, in *in vitro* systems using mouse macrophages or human monocytes.

In this context, a thorough investigation is needed to examine the epigenetic and metabolic profiles induced in monocytes/macrophages by NP exposure and correlate them to functional memory profiles. Thus, both the immunosafety of NP and their medical exploitation have to be re-considered in terms of induction of epigenetic and metabolic changes and of induction/regulation of innate memory. This information could pave the way to new nanotechnological applications in medicine.

## Author Contributions

PI wrote the manuscript. GDC contributed to writing. DB revised the article. All authors contributed to the article and approved the submitted version.

## Conflict of Interest

The authors declare that the research was conducted in the absence of any commercial or financial relationships that could be construed as a potential conflict of interest.
